# Survival outcomes of intrathoracic vs. cervical anastomosis post-esophagectomy in middle and lower thoracic esophageal squamous cell carcinoma: a retrospective propensity score matching analysis

**DOI:** 10.3389/fonc.2025.1632594

**Published:** 2025-10-14

**Authors:** Xueqiang Wei, Jie Mao, Yuncheng Bai, Hao Yang, Yizhou Peng, Jin Liu, Zhenghai Shen, Shengguai Gao, Huiqiao Wang, Xiaobo Chen, Ying Chen, Jiapeng Yang, Yunchao Huang

**Affiliations:** ^1^ Department of Thoracic and Cardiovascular Surgery, The Third Affiliated Hospital of Kunming Medical University (Yunnan Tumor Hospital), Kunming, China; ^2^ Department of Orthopedic Surgery, The First People’s Hospital of Yunnan Province, Affiliated Hospital of Kunming University of Science and Technology, Kunming, China

**Keywords:** esophageal squamous cell carcinoma, cervical anastomosis, intrathoracic anastomosis, esophagectomy, overall survival, propensity score matching

## Abstract

**Objective:**

This study aimed to compare long-term survival outcomes between cervical anastomosis (CA) and intrathoracic anastomosis (IA) in patients with middle and lower thoracic esophageal squamous cell carcinoma (ESCC).

**Methods:**

A retrospective cohort analysis was conducted on 571 patients who underwent esophagectomy at a single institution. Patients were stratified into CA and IA groups based on anastomotic technique. Propensity score matching (PSM, 1:1) was applied to balance baseline covariates. Overall survival (OS) and disease-free survival (DFS) were evaluated using Kaplan-Meier analysis and Cox regression. Secondary outcomes included postoperative complications.

**Results:**

In the unmatched cohort, CA demonstrated superior OS (median: 51.17 vs. 34.50 months; HR: 1.368, 95% CI: 1.062–1.763; p=0.015) and DFS (median: 45.07 vs. 28.87 months; HR: 1.289, 95% CI: 1.013–1.641; p=0.039) compared to IA. However, after PSM, the survival advantage attenuated (OS: HR = 1.303, 95% CI: 0.953–1.780, p=0.097; DFS: HR = 1.295, 95% CI: 0.962–1.744, p=0.089). Multivariate analysis identified pathological T3/T4 stages (OS: p=0.002–0.009; DFS: p<0.001) and lymphovascular invasion (DFS: p=0.023) as dominant prognostic factors, overshadowing anastomotic technique. The CA group exhibited more extensive lymph node dissection (>7 stations, p<0.001), but short-term mortality (30-/90-day) did not differ between groups (p≥0.382).

**Conclusion:**

In conclusion, our study suggests that there may be a potential survival advantage of CA over IA in patients undergoing esophagectomy for ESCC. However, the initial survival benefits associated with CA diminished after adjusting for confounding factors.

## 1 Introduction

Esophageal cancer (EC) ranks as the eighth most prevalent malignancy and the sixth leading cause of cancer-related mortality worldwide. It exhibits significant geographic heterogeneity, with East Asia—particularly China—bearing the highest burden of esophageal squamous cell carcinoma (ESCC) (1–4). In China, ESCC accounts for over half of global cases, characterized by late-stage diagnoses and poor prognoses, making it the seventh most prevalent and fifth most lethal cancer nationally (1–4). The survival of patients with esophageal cancer is influenced by numerous factors, including surgical details such as lymph node resection, which are crucial for determining long-term prognosis (5–9). Despite advancements in multimodal therapies that combine surgery, radiotherapy, chemotherapy, and immunotherapy, long-term survival for patients with ESCC remains suboptimal. This underscores the critical role of esophagectomy as the cornerstone of curative intent (9–13).

The surgical management of ESCC necessitates esophageal resection followed by reconstruction via anastomosis, with intrathoracic (IA) and cervical (CA) techniques representing the primary approaches. The choice between these methods remains contentious, as each carries distinct trade-offs. Proponents of IA emphasize its technical feasibility, robust vascular supply, and reduced anastomotic leakage risk, whereas CA advocates highlight its potential for wider resection margins and lower locoregional recurrence, possibly conferring survival advantages (14–19). However, conflicting evidence persists: recent studies associate IA with fewer complications like recurrent laryngeal nerve injury, while emerging propensity-adjusted analyses suggest CA may improve overall survival, even after confounder adjustment. These survival benefits, however, must be balanced against CA’s higher morbidity rates, including anastomotic stenosis and pneumonia (14–19).

This study evaluates long-term survival following IA versus CA in middle and lower thoracic ESCC through a retrospective analysis, focusing on survival evidence. Our findings aim to inform surgical decision-making.

## 2 Materials and methods

### 2.1 Data source

**Figure 1:**
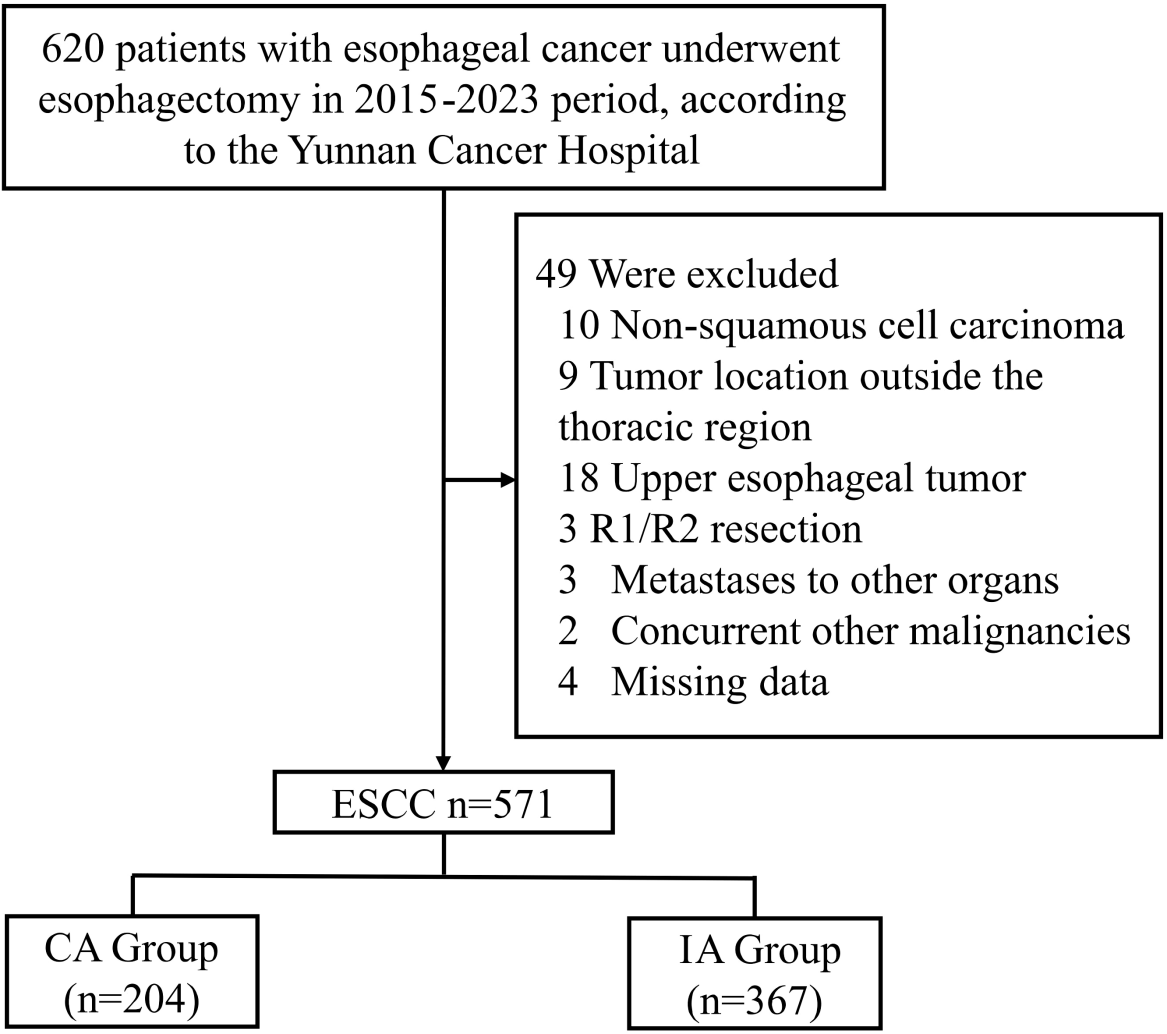
CONSORT diagram of patient selection. ESCC, esophageal squamous cell carcinoma; IA, intrathoracic anastomosis; CA, cervical anastomosis.

This study was a retrospective cohort analysis, designed to evaluate long-term survival outcomes following two different surgical approaches for middle and lower thoracic ESCC: CA and IA. Patients was conducted using data from Yunnan Cancer Hospital who underwent esophagectomy from January 2015 to December 2023. The primary endpoints of the study were OS and DFS. OS was defined as the time from the initiation of the treatment to the date of death from any cause or the last follow-up, while DFS was defined as the time from the initiation of the treatment to the first occurrence of disease recurrence, death, or the last follow-up, whichever came first. Inclusion criteria for the study were as follows (1): diagnosed with ESCC under esophagectomy (2), available demographic, clinical, and pathological data. Exclusion criteria were (1): patients diagnosed with non-squamous cell esophageal cancer (2), esophageal tumors located in the upper thoracic region (3), incomplete resection (4), presence of distant metastasis at the time of diagnosis (5), missing data. A total of 591 patients met the inclusion criteria and were enrolled in the study (**Figure 1**). All patients underwent esophagectomy performed by experienced surgeons specializing in thoracic and cardiovascular surgery. Depending on the location of the anastomosis, patients were classified into two groups: cervical anastomosis (CA group), and intrathoracic anastomosis (IA group). The surgical procedures for both approaches followed standard protocols for esophagectomy, including extensive lymph node dissection, careful resection of the tumor, and reconstruction via anastomosis to restore gastrointestinal continuity. The choice between CA and IA was made based on preoperative evaluation, tumor location, surgeon preference, and other clinical factors. Demographic data collected included age, gender and so on. Patients were followed up regularly postoperatively, typically every 3 months for the first two years and then every 6 months thereafter, or until the most recent follow-up. Follow-up included clinical evaluation, imaging studies (chest CT, upper GI endoscopy), and pathological assessments if required. The primary outcome measures were overall survival (OS) and disease-free survival (DFS). Secondary outcomes included postoperative complications such as anastomotic leakage, recurrent laryngeal nerve injury, pneumonia, and the incidence of anastomotic stenosis. CA patients routinely underwent the McKeown procedure, while IA patients were all treated using the Ivor-Lewis procedure, without any special modifications.

### 2.2 Ethical statement

This study was conducted in accordance with the Declaration of Helsinki (2013 revision). This study was conducted in compliance with ethical standards and received approval from the Ethics Committee for Medical Research and New Medical Technology of Yunnan Cancer Hospital (Approval No. KYCS2025-176).

### 2.3 Statistical analysis

Continuous variables were expressed as means and standard deviations, while categorical variables were presented as frequencies and percentages. To compare the differences between the CA and IA groups, independent t-tests were performed for continuous variables, while chi-square or Fisher’s exact tests were used for categorical variables, depending on the data distribution. The Kaplan-Meier method was employed to estimate the OS and DFS curves, and the log-rank test was used to compare these survival outcomes between the two groups. To account for potential confounding variables and minimize selection bias, propensity score matching (PSM) was applied. The propensity scores were calculated using a logistic regression model, where the treatment approach (CA vs. IA) was the dependent variable, and baseline covariates such as age, sex, and other significant clinical characteristics were included as independent variables. A 1:1 nearest-neighbor matching algorithm without replacement was employed. After matching, covariate balance between the two groups was assessed using absolute mean differences (AMD), with an AMD value below 0.4 indicating satisfactory balance between the groups. Univariable and multivariable Cox proportional hazards regression models were used to identify independent risk factors associated with OS and DFS. Hazard ratios (HRs) with 95% confidence intervals (CIs) were reported. The multivariable models were adjusted for potential confounders identified through the propensity score matching, ensuring that the HRs and CIs were adjusted for these variables. To further refine the analysis, additional weighting approaches were used to adjust for potential confounders, and the adjusted HRs with corresponding 95% CIs were calculated. All statistical analyses were conducted using SPSS software (version 25.0, IBM Corp, Armonk, NY, USA) and RStudio with R version 4.3.0. A two-sided p-value of less than 0.05 was considered statistically significant for all analyses.

## 3 Results

### 3.1 Clinical outcomes

A total of 571 patients who met the inclusion criteria were analyzed in this study (**Figure 1**). Among the included patients, 551 (96.50%) were male, and 20 (3.50%) were female. The age distribution showed that 314 patients (54.99%) were below 60 years, while 257 patients (45.01%) were aged 60 years or older. A significant proportion of the cohort had histories of smoking (429 patients, 75.13%) and heavy drinking (426 patients, 74.60%). In terms of clinical TNM staging, 332 patients (58.14%) were classified as stage III or IV, and 426 (74.60%) underwent neoadjuvant therapy prior to surgery (**Table 1**). Postoperative pathological findings revealed that 149 patients (26.09%) had tumors graded as poor or undifferentiated (G3-4). Lymphovascular invasion was observed in 52 patients (9.10%), and nerve invasion was present in 40 patients (7.01%). Pathological TNM staging identified 306 patients (53.60%) in stage III or IV, consistent with the advanced stage of the disease in the majority of the cohort (**Table 2**). Significant differences were observed between the CA and IA groups regarding the extent of lymph node stations dissection. Specifically, the number of patients with more than seven lymph node stations dissected was significantly higher in the CA group compared to the IA group (p < 0.001). However, no significant differences were found between the CA and IA groups in terms of short-term postoperative mortality, including deaths within 30 days (p = 0.405) or 90 days (p = 0.382) post-surgery (**Table 2**).

**Table 1:** Demographic characteristics of the 2 groups.

Characteristic		Before PSM		*P* value	After PSM		*P* value
	Total (n=571)	CA (n=204)	IA (n=367)		CA (n=162)	IA (n=162)	
Sex				0.378			1.000
Male	551 (96.50%)	195 (95.59%)	356 (97.00%)		156 (96.30%)	156 (96.30%)	
Female	20 (3.50%)	9 (4.41%)	11 (3.00%)		6 (3.70%)	6 (3.70%)	
Age, years				0.058			0.309
median (range)	58 (36–80)	58 (36–79)	59(37-80)		58 (39-79)	57(37-80)	
<60	314 (54.99%)	123 (60.29%)	191 (52.04%)		91 (56.17%)	100 (61.73%)	
≥60	257 (45.01%)	81 (39.71%)	176 (47.96%)		71 (43.83%)	62 (38.27%)	
Smoking				0.205			0.900
Yes	429 (75.13%)	147 (72.06%)	282 (76.84%)		120 (74.07%)	119 (73.46%)	
No	142 (24.87%)	57 (27.94%)	85 (23.16%)		42 (25.93%)	43 (26.54%)	
Alcohol				0.872			0.256
Yes	426 (74.61%)	153 (75.00%)	273 (74.39%)		124 (76.54%)	115 (70.99%)	
No	145 (25.39%)	51 (25.00%)	94 (25.61%)		38 (23.46%)	47 (29.01%)	
BMI				0.429			0.884
Low	108 (18.91%)	35 (17.16%)	73 (19.89%)		30 (18.52%)	29 (17.90%)	
Normal	360 (63.05%)	127 (62.25%)	233 (63.49%)		101 (62.35%)	105 (64.81%)	
High	103 (18.04%)	42 (20.59%)	61 (16.62%)		31 (19.14%)	28 (17.28%)	
KPS score				0.456			0.945
70	10 (1.75%)	5 (2.45%)	5 (1.36%)		4 (2.47%)	4 (2.47%)	
80	141 (24.69%)	46 (22.55%)	95 (25.89%)		41 (25.31%)	44 (27.16%)	
90	420 (73.56%)	153 (75.00%)	267 (72.75%)		117 (72.22%)	114 (70.37%)	
Neoadjuvant therapy				<0.001			0.473
Yes	426 (74.61%)	108 (52.94%)	318 (86.65%)		108 (66.67%)	114 (70.37%)	
No	145 (25.39%)	96 (47.06%)	49 (13.35%)		54 (33.33%)	48 (29.63%)	
Tumor location				<0.001			0.261
Middle	218 (38.18%)	115 (56.37%)	103 (28.07%)		74 (45.68%)	64 (39.51%)	
Lower	353 (61.82%)	89 (43.63%)	264 (71.93%)		88 (54.32%)	98 (60.49%)	
Clinical T stage				0.589			0.680
T1	5 (0.99%)	2 (0.98%)	3 (0.82%)		1 (0.62%)	1 (0.62%)	
T2	193 (33.80%)	63 (30.88%)	130 (35.42%)		59 (36.42%)	49 (30.25%)	
T3	328 (57.44%)	120 (58.82%)	208 (56.68%)		86 (53.09%)	97 (59.88%)	
T4	45 (7.88%)	19 (9.31%)	26 (7.08%)		16 (9.88%)	15 (9.26%)	
Clinical N stage				0.270			0.130
N0	204 (35.73%)	66 (32.35%)	138 (37.60%)		63 (38.89%)	50 (30.86%)	
N1	137 (23.99%)	45 (22.06%)	92 (25.07%)		35 (21.60%)	48 (29.63%)	
N2	244 (39.23%)	91 (44.61%)	133 (36.24%)		62 (38.27%)	64 (39.51%)	
N3	6 (1.05%)	2 (0.98%)	4 (1.09%)		2 (1.23%)	0 (0.00%)	
Clinical 8th TNM Stage				0.483			0.454
I	4 (0.70%)	1 (0.49%)	3 (0.82%)		1 (0.62%)	1 (0.62%)	
II	235 (41.16%)	76 (37.25%)	159 (43.32%)		70 (43.21%)	59 (36.42%)	
III	281 (49.21%)	106 (51.96%)	175 (47.68%)		73 (45.06%)	87 (53.70%)	
IV	51 (8.93%)	21 (10.29%)	30 (8.17%)		18 (11.11%)	15 (9.26%)	

KPS, Karnofsky Performance Status; PSM, propensity score matching; TNM, tumor, node, metastasis.

**Table 2:** Demographic characteristics of the 2 groups after surgery.

Characteristic		Before PSM		*P* value	After PSM		*P* value
	Total (n=571)	CA (n=204)	IA (n=367)		CA (n=162)	IA (n=162)	
Pathological differentiation grade				<0.001			0.497
No tumor cells.	22 (3.85%)	17 (8.33%)	5 (1.36%)		8 (4.94%)	4 (2.47%)	
Moderate or Well G1-2	400 (70.05%)	138 (67.65%)	262 (71.39%)		111 (68.52%)	113 (69.75%)	
Poor or undifferentiated G3-4	149 (26.09%)	49 (24.02%)	100 (27.25%)		43 (25.54%)	45 (27.78%)	
Lymphovascular invasion				<0.001			0.220
Yes	52 (9.11%)	21 (10.29%)	31 (8.45%)		16 (9.88%)	10 (6.17%)	
No	519 (90.89%)	183 (89.71%)	336 (91.55%)		146 (90.12%)	152 (93.83%)	
Nerve invasion				0.204			0.279
Yes	40 (7.01%)	18 (8.82%)	22 (5.99%)		14 (8.64%)	9 (5.56%)	
No	531 (92.99%)	186 (91.18%)	345 (94.01%)		148 (91.36%)	153 (94.44%)	
Pathological T stage				<0.001			0.018
T0	22 (3.85%)	17 (8.33%)	5 (1.36%)		8 (4.94%)	4 (2.47%)	
T1	87 (15.24%)	39 (19.12%)	48 (13.08%)		35 (20.60%)	21 (12.96%)	
T2	132 (23.12%)	61 (29.90%)	71 (19.35%)		50 (30.86%)	38 (23.46%)	
T3	327 (57.27%)	85 (41.67%)	242 (65.94%)		68 (41.98%)	98 (60.49%)	
T4	3 (0.53%)	2 (0.98%)	1 (0.27%)		1 (0.62%)	1 (0.62%)	
Pathological N stage				0.796			0.323
N0	255 (44.66%)	95 (46.57%)	160 (43.60%)		76 (46.91%)	74 (45.68%)	
N1	183 (32.05%)	60 (29.41%)	123 (33.51%)		43 (26.54%)	56 (34.57%)	
N2	98 (17.16%)	36 (17.65%)	62 (16.89%)		32 (19.75%)	25 (15.43%)	
N3	35 (6.13%)	13 (6.37%)	22 (5.99%)		11 (6.79%)	7 (4.32%)	
Pathological 8th TNM Stage				0.102			0.129
I	95 (16.64%)	51 (25.00%)	44 (11.99%)		37 (22.84%)	23 (14.20%)	
II	170 (29.77%)	47 (23.04%)	123 (33.51%)		42 (25.93%)	53 (32.72%)	
III	268 (46.94%)	91 (44.61%)	177 (48.23%)		71 (43.83%)	78 (48.15%)	
IV	38 (6.65%)	15 (7.35%)	23 (6.27%)		12 (7.41%)	8 (4.94%)	
No. of RLN stations				<0.001			<0.001
<7	355 (62.17%)	91 (44.61%)	264 (71.93%)		81 (50.00%)	120 (74.07%)	
≥7	216 (37.83%)	113 (55.39%)	103 (28.07%)		81 (50.00%)	42 (25.93%)	
Died in 30 days	4 (0.70%)	3 (1.47%)	1 (0.27%)	0.133	1 (0.62%)	0 (0.00%)	0.317
Died in 90 days	14 (2.45%)	12 (5.88%)	2 (0.54%)	<0.001	4 (2.47%)	1 (0.62%)	0.371

KPS, Karnofsky Performance Status; PSM, propensity score matching; TNM, tumor, node, metastasis; No. of RLN stations, number of resected lymph node stations.

### 3.2 Survival outcomes

**Figure 2:**
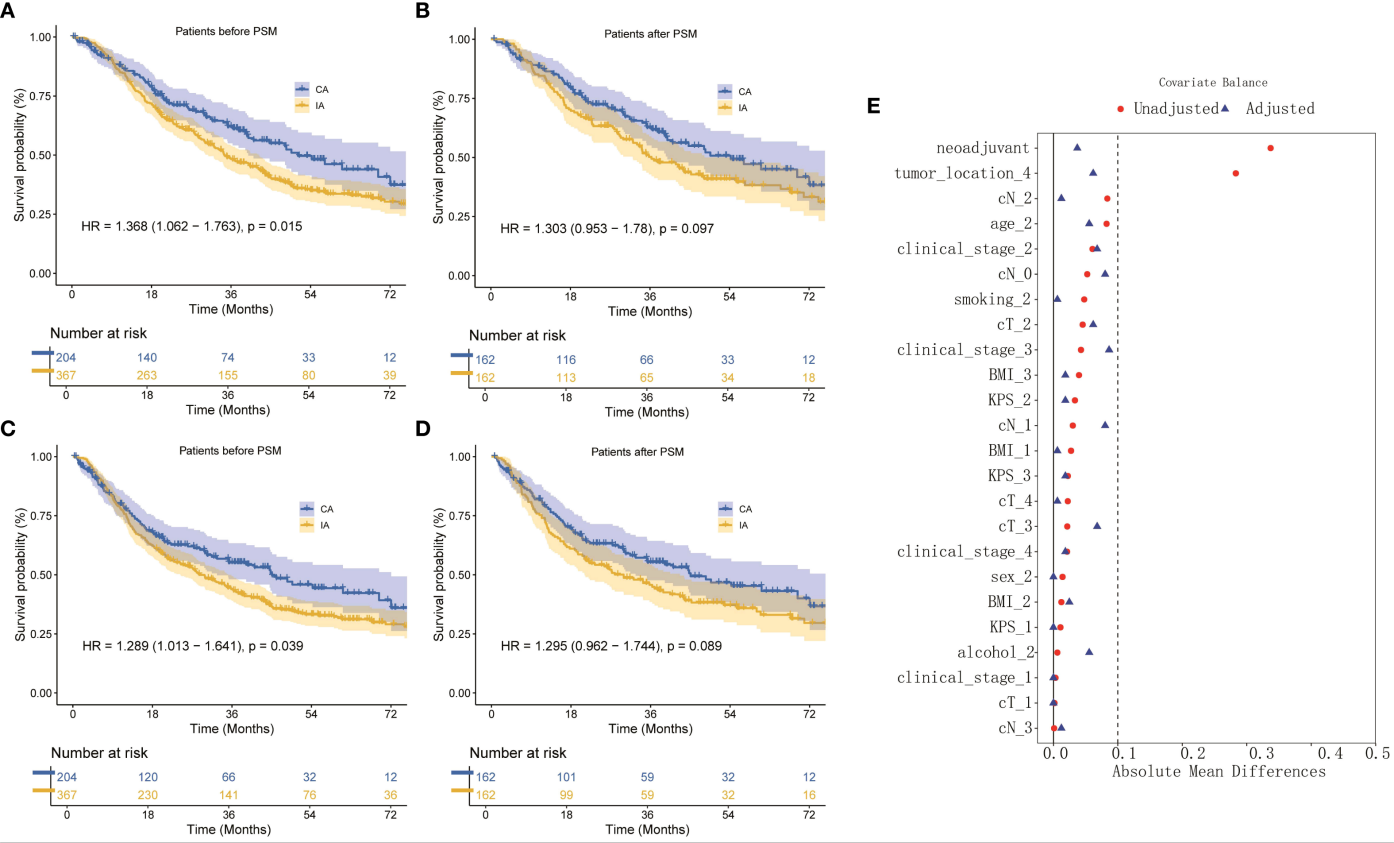
**(A)** OS curve of CA and IA groups, **(B)** OS curve of CA and IA groups after PSM, **(C)** DFS curve of CA and IA groups, **(D)** DFS curve of CA and IA groups after PSM, **(E)** Absolute in the subjects stratified by characteristic.

The median follow-up duration for the cohort was 54.27 months. In terms of OS, patients in the CA group had a median OS time of 51.17 months (95% CI: 37.77–65.56), compared to 34.50 months (95% CI: 28.59–40.14) for those in the IA group. The one-year OS rate for the CA group was 87%, with three-year and five-year rates at 62% and 46%, respectively. In contrast, the IA group exhibited a one-year OS rate of 85%, a three-year rate of 49%, and a five-year rate of 34% (HR: 1.368; 95% CI: 1.062–1.763; P = 0.015; **Figure 2A**). After propensity score matching, the OS advantage of the CA group over the IA group persisted, although the difference was not statistically significant (HR: 1.303; 95% CI: 0.953–1.780; P = 0.097; **Figure 2B**). Regarding DFS, the CA group demonstrated a median DFS time of 45.07 months (95% CI: 30.56–59.58), while the IA group showed a median DFS time of 28.87 months (95% CI: 23.19–34.54). The DFS rates for the CA group were 78% at one year, 55% at three years, and 44% at five years. For the IA group, the DFS rates were 76% at one year, 44% at three years, and 32% at five years (HR: 1.289; 95% CI: 1.013–1.641; P = 0.039; **Figure 2C**). Following propensity score matching, the CA group continued to show better DFS than the IA group; however, the difference was not statistically significant (HR: 1.295; 95% CI: 0.962–1.744; P = 0.089; **Figure 2D**). **Figure 2E** illustrated that for 1:1 PSM, the AMD for all variables is less than 0.1.

### 3.3 Short-term outcomes

The results of short-term outcomes indicate that, except for Hydrothorax, there were no significant differences between the groups before and after Propensity Score Matching (PSM). Before PSM, 32 patients (15.69%) in the CA group and 31 patients (8.45%) in the IA group developed Hydrothorax (P = 0.008), with the CA group showing a significantly higher rate of Hydrothorax compared to the IA group. After PSM, although the difference decreased (P = 0.066), the CA group still had a higher incidence of Hydrothorax compared to the IA group (26 patients, 16.05% vs. 15 patients, 9.26%) (**Table 3**).

**Table 3:** Adverse events.

Adverse events	Before PSM			After PSM		
	CA (n=204)	IA (n=367)	P value	CA (n=162)	IA (n=162)	P value
Anastomotic stenosis	5(2.45%)	19(5.18%)	0.120	4(2.47%)	7(4.32%)	0.357
Anastomotic leakage	11(5.39%)	25(6.81%)	0.504	7(4.32%)	11(6.79%)	0.332
Pulmonary infection	27(13.24%)	35(9.54%)	0.173	18(11.11%)	13(8.02%)	0.343
Hydrothorax	32(15.69%)	31(8.45%)	0.008	26(16.05%)	15(9.26%)	0.066
Respiratory failure	8(3.92%)	10(2.72%)	0.433	6(3.70%)	5(3.09%)	0.759
Heart failure	1(0.49%)	7(1.91%)	0.270	0(0.00%)	4(2.47%)	0.123
Pneumothorax	7(3.43%)	4(1.09%)	0.061	6(3.70%)	3(1.85%)	0.502

### 3.4 Risk factors

**Figure 3:**
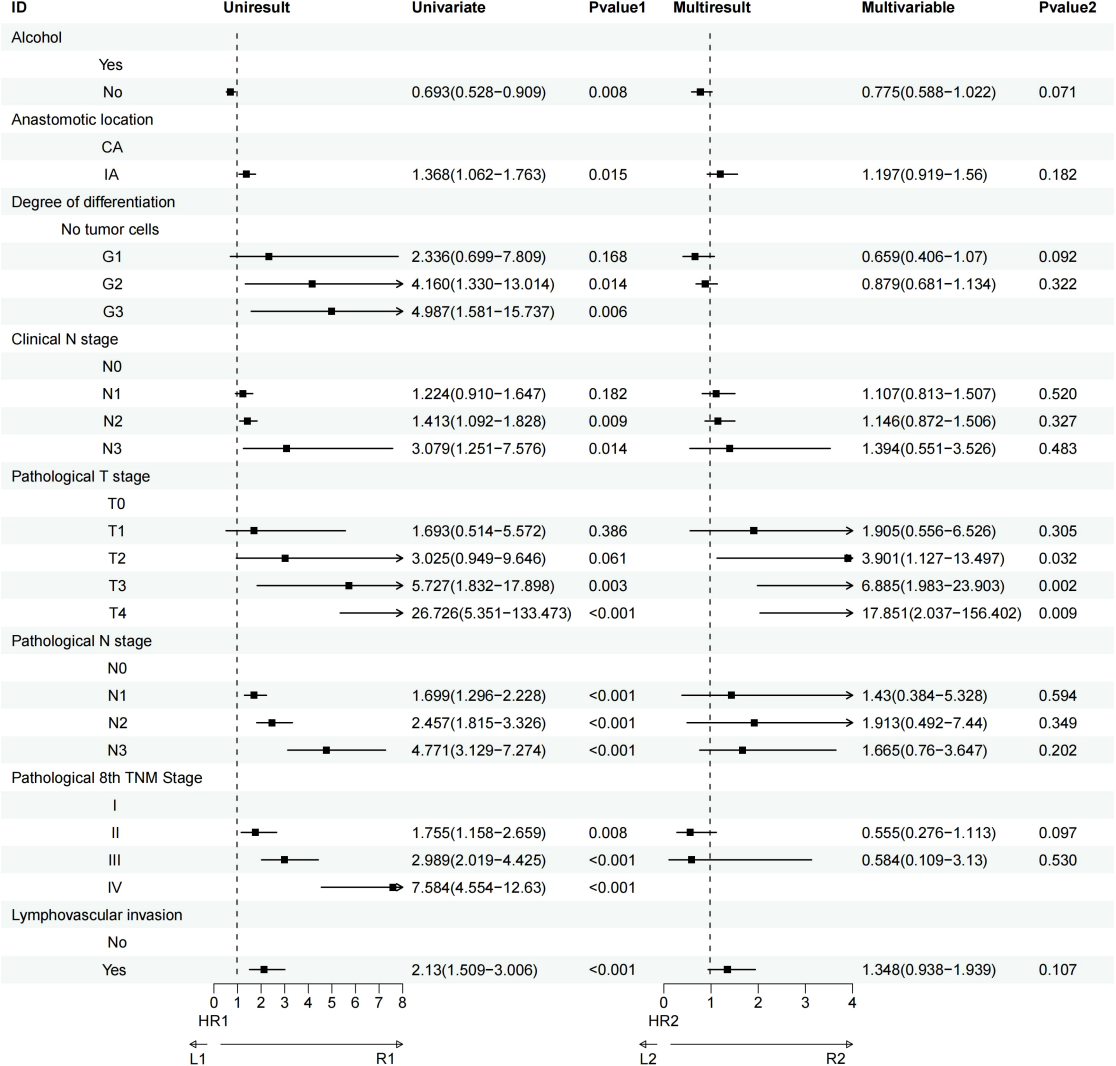
Univariate and multivariate Cox regression analyses of factors affecting patient OS.

**Figure 4:**
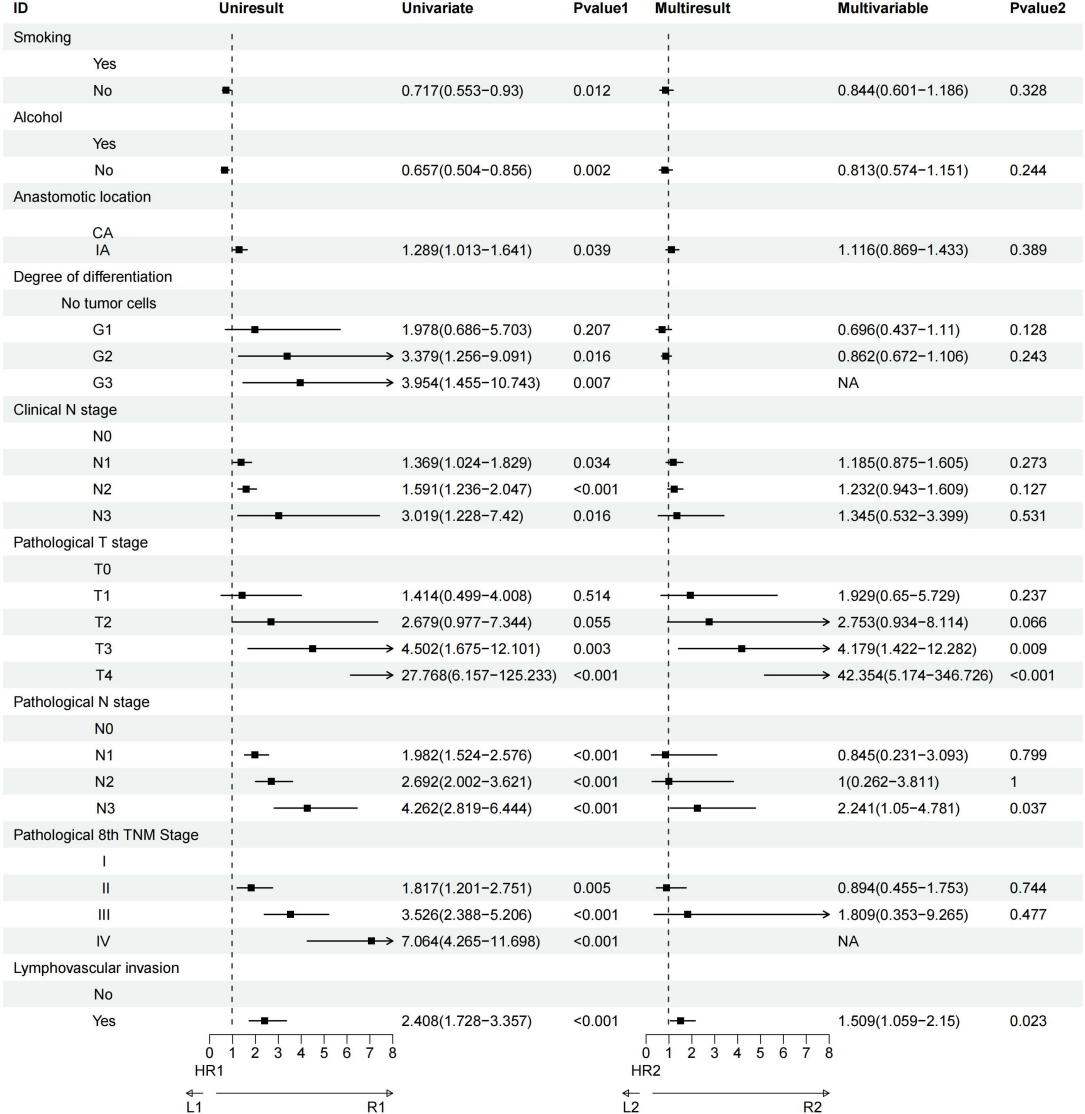
Univariate and multivariate Cox regression analyses of factors affecting patient DFS.

Univariate analysis revealed that factors such as a history of heavy drinking, anastomotic location, degree of tumor differentiation, clinical N stage, pathological T and N stages, pathological TNM stage, and lymphovascular invasion significantly influenced OS. Further multivariate Cox regression analysis identified pathological T3 stage (P = 0.002) and pathological T4 stage (P = 0.009) as the most critical determinants of OS (**Figure 3**). Similarly, univariate analysis demonstrated that smoking history, heavy drinking, anastomotic location, tumor differentiation, clinical N stage, pathological T and N stages, pathological TNM stage, and lymphovascular invasion were significant factors affecting DFS. Multivariate Cox regression further highlighted that pathological T3 stage (P = 0.009), pathological T4 stage (P<0.001), pathological N3 stage (P = 0.037), and lymphovascular invasion (P = 0.023) were the most influential prognostic factors for DFS (**Figure 4**).

## 4 Discussion

This retrospective cohort study compared long-term survival outcomes between CA and IA in patients with middle and lower thoracic ESCC. The results demonstrated that CA was associated with superior OS and DFS compared to IA in the overall cohort. However, after PSM to adjust for baseline confounders, these survival advantages were attenuated and no longer statistically significant.

The observed OS and DFS benefits for CA in the pre-matched analysis align with prior studies suggesting that CA may facilitate wider resection margins and more extensive lymph node dissection, potentially reducing locoregional recurrence and improving oncologic outcomes (14–16). Our data revealed that the CA group underwent dissection of significantly more lymph node stations (>7) compared to IA, which may partially explain its initial survival advantage. Notably, pathological T3/T4 stages and lymphovascular invasion were independently associated with poorer OS and DFS. These findings emphasize that advanced tumor invasion and aggressive biological behavior outweigh the impact of anastomotic technique on survival.

The prognosis of ESCC is influenced by multiple, potentially overlapping factors (20–24). While the current standard of care prioritizes surgical resection following neoadjuvant therapy, surgical variables remain critical determinants of outcomes (25–28). Our study investigated the impact of anastomotic location on survival, with findings suggesting that the observed differences may fundamentally stem from variations in lymph node dissection. Notably, the CA group demonstrated a significantly higher proportion of lymph node stations resected (>7 stations) compared to the IA group—a finding consistent with prior studies (15, 17). Although PSM attenuated survival differences and CA showed no statistically significant survival advantage over IA in matched cohorts, a clinically relevant gap in OS and DFS persisted between the groups. This trend suggests that prolonged follow-up and larger patient cohorts may amplify these differences, potentially revealing clearer survival benefits for CA over time. The survival gap observed in the unmatched analysis aligns with the hypothesis that CA facilitates more extensive lymphadenectomy, thereby addressing occult micrometastases and reducing locoregional recurrence (15–19).

When contextualized with the meta-analysis by You et al., highlight a critical tension in surgical decision-making for ESCC: the balance between survival outcomes and postoperative morbidity. While our unmatched analysis initially suggested a survival advantage for CA, the attenuation of this benefit after PSM underscores the confounding influence of baseline factors such as lymph node dissection extent and tumor biology. However, IA’s lower complication rates—particularly for anastomotic leak (RR = 2.76 for CA in You et al.)—may mitigate its theoretical survival disadvantages by reducing delays in adjuvant therapy and preserving quality of life (29).

The integration of findings from our study similar to work by Li et al. provides a nuanced perspective on the relationship between anastomotic location, lymphadenectomy extent, and survival outcomes in ESCC. While both studies highlight the potential survival advantages of CA, the mechanisms underlying these benefits appear intrinsically tied to the extent of lymph node dissection, particularly in the upper mediastinal zone. Notably, Li et al. identified significantly more extensive dissection of recurrent laryngeal nerve (RLN)-associated LN stations (105, 106recL/R) in the CA group, which correlated with reduced upper mediastinal recurrence (7.1% vs. 15.7%, P<0.001) and LN recurrence (19.1% vs. 28.4%, P<0.001). These results suggest that CA facilitates superior en bloc resection of occult micrometastases in the RLN region, a common site for ESCC recurrence (15).

These collective findings highlight the critical interplay between surgical technique, anatomical access, and oncologic precision in ESCC management. The superior upper mediastinal lymph node dissection achieved through CA—particularly in high-risk zones like the RLN basins—likely disrupts metastatic pathways that IA approaches may incompletely address, given their technical constraints in visualizing and resecting supra-aortic arch structures. From an evolutionary surgical perspective, these results advocate for a paradigm shift from binary technique comparisons to quality-oriented metrics, particularly standardized lymphadenectomy templates incorporating bilateral RLN node dissection. Future trials should stratify by both anastomotic approach and achieved lymph node yield to disentangle their individual contributions while accounting for the learning curve effect, as CA’s technical demands may introduce performance bias in non-specialized centers.

It is important to explore whether IA combined with postoperative radiotherapy (PORT), specifically targeting the upper mediastinal nodes, could offer comparable local control to CA in patients who are unfit for extensive lymphadenectomy, such as elderly patients or those with poor pulmonary function. IA combined with PORT may provide an alternative approach to achieve better local control in patients who cannot undergo aggressive lymph node dissection due to contraindications such as compromised health or anatomical considerations. The use of PORT in such cases may help to target residual microscopic disease in the mediastinal region, potentially improving local control without the need for extensive lymphadenectomy. It is essential to consider the balance between potential benefits and the risks associated with radiation therapy in these vulnerable populations. Future studies could further clarify whether this combination approach offers comparable long-term outcomes in terms of local control, DFS, and OS, particularly for patients who are not candidates for comprehensive surgical interventions. By incorporating this consideration, personalized decision-making can be better guided, optimizing treatment strategies for patients based on their individual health status and disease characteristics.

This study has several limitations inherent to its retrospective design. First, unmeasured confounders, such as surgeon experience, precise tumor location, and adjuvant therapy adherence, may have influenced outcomes. Second, the single-center design and modest sample size post-PSM limit generalizability, potential bias from single-center surgical expertise homogeneity (e.g., whether CA/IA selection was influenced by surgeon experience) and its impact on generalizability. Third, the median follow-up of 54 months, while substantial, may not capture very late recurrences. Then, our study lacks a detailed classification of lymph nodes by individual stations. This limitation arises from the use of the NCCN guidelines for staging in the pathology reports, which typically categorize lymph node involvement into broader groups without distinguishing between individual stations. Finally, the absence of standardized criteria for selecting CA versus IA introduces residual selection bias, despite PSM adjustment.

## 5 Conclusions

This retrospective cohort study found middle and lower thoracic ESCC, CA was initially associated with improved OS and DFS compared to IA, but this advantage diminished after balancing baseline covariates.

## Data Availability

The raw data supporting the conclusions of this article will be made available by the authors, without undue reservation.
